# Enzymatization of mouse monoclonal antibodies to the corresponding catalytic antibodies

**DOI:** 10.1038/s41598-024-63116-6

**Published:** 2024-05-28

**Authors:** Emi Hifumi, Yuina Ito, Moe Tsujita, Hiroaki Taguchi, Taizo Uda

**Affiliations:** 1 Institute for Research Management, Oita University, 700 Dannoharu, Oita-shi, Oita 870-1192 Japan; 2 Research Center for GLOBAL/LOCAL Infectious Diseases, Oita University, 700 Dannoharu, Oita-shi, Oita 870-1192 Japan; 3 Graduate School of Engineering, Oita University, 700 Dannoharu, Oita-shi, Oita 870-1192 Japan; 4 Faculty of Pharmaceutical Sciences, Suzuka University of Medical Science, 3500-3 Minamitamagaki-cho, Suzuka, 510-0293 Japan; 5 Materials Open Laboratory, Institute of Systems, Information Technologies and Nanotechnologies (ISIT), Fukuoka, 819-0388 Japan

**Keywords:** Monoclonal antibody, Influenza virus, Hemagglutinin, Catalytic antibody, Förster resonance energy transfer substrate, Biochemistry, Biotechnology, Immunology, Molecular biology

## Abstract

Catalytic antibodies possess a dual function that enables both antigen recognition and degradation. However, their time-consuming preparation is a significant drawback. This study developed a new method for quickly converting mice monoclonal antibodies into catalytic antibodies using site-directed mutagenesis. Three mice type monoclonal antibodies targeting hemagglutinin molecule of influenza A virus could be transformed into the catalytic antibodies by deleting Pro95 in CDR-3 of the light chain. No catalytic activity was observed for monoclonal antibodies and light chains. In contrast, the Pro95-deleted light chains exhibited a catalytic activity to cleave the antigenic peptide including the portion of conserved region of hemagglutinin molecule. The affinity of the Pro95-deleted light chains to the antigen increased approximately 100-fold compared to the wild-type light chains. In the mutants, three residues (Asp1, Ser92, and His93) come closer to the appropriate position to create the catalytic site and contributing to the enhancement of both catalytic function and immunoreactivity. Notably, the Pro95-deleted catalytic light chains could suppress influenza virus infection in vitro assay, whereas the parent antibody and the light chain did not. This strategy offers a rapid and efficient way to create catalytic antibodies from existing antibodies, accelerating the development for various applications in diagnostic and therapeutic applications.

## Introduction

Since the natural catalytic antibody was found in 1989, many studies on the catalytic antibodies have been carried out to date. They have unique features not only to recognize the antigen but also to decompose it. Interestingly, they enzymatically degrade antigens, such as peptides^1–5^, antigenic proteins^6–11^, DNA^12–14^, and physiologically active molecules^15–19^. Along with these basic studies, interesting applications of catalytic antibodies have been reported so far. The catalytic antibody UA-15L degrading *H. pylori* urease possesses the ability not only to degrade the β-subunit of the urease but also eliminate the enzymatic activity of the urease. In addition, oral administration of UA15-L significantly reduced the number of infected *H. pylori* in mice stomach^20^. Durova et al. developed a “catalytic vaccine” against HIV-1 gp120. Its function exceeds that of classical antibody^21^. For the application to agricultural field, Smirnov et al. developed the unique catalytic antibody, which can detoxify the organophosphate pesticide paraoxon. They named the catalytic antibody as “Reactibody”^22^. Regarding the study on rabies virus, catalytic antibody light chain designated as #18 clone could suppress the infection of rabies virus. Notably, the survival rate of mice in which a lethal level of the rabies virus was co-inoculated directly into the brain with catalytic antibody light chain was significantly improved^23^. Amyloid-β (Aβ) is well known one of the molecules causing Alzheimer disease. Paul et al. succeeded in the clearance of Aβ aggregates depositing on brain of mice by using anti Aβ catalytic antibody^24^. Study on safety tests of catalytic antibody was also done. The acute toxic features of the catalytic antibody light chain were investigated. Multiple doses of 8.3 mg/kg were given to mice by intravenous inoculation every seven days. No remarkable appearances or occurrences were observed in body weight, food and water intake, and dissection. This suggests that catalytic antibody can be used safely^25^. These interesting and useful features should be directed to further development in medical fields in addition to diagnosis and agriculture fields as the novel enzymes.

Monoclonal catalytic antibodies can be obtained in several steps, including antigen design, immunization, antibody production, and antibody screening. This preparation method requires considerable time and effort, and its inconvenience makes the application of catalytic antibodies challenging in various fields. Namely, a major drawback exists in the production of the desired catalytic antibodies. Developing an evolutionary production method is necessary in which the catalytic antibody can be prepared in a short time and an easy manner. To overcome this issue, a promising algorithm has been reported in which the direct conversion of a human monoclonal antibody (mAb) to the corresponding catalytic antibody was enabled by deleting Pro95 (or neighboring Pro residue) in the CDR-3 in the light chain^26^. Using this method, some human antibodies were converted into catalytic antibodies that exhibited enzymatic functions to cleave antigen molecules^27^. To date, thousands of mice mAbs have been developed in the world since 1975^28^. Conversion of these mAbs into catalytic antibodies would help in obtaining desired catalytic antibodies without the challenges mentioned above. To realize this concept, we investigated whether mice mAbs could be easily converted to the corresponding catalytic antibodies in a short time using the new algorithm^26,27^.

## Results

### Mice mAbs and the amino acid (aa) sequences

**Figure 1:**
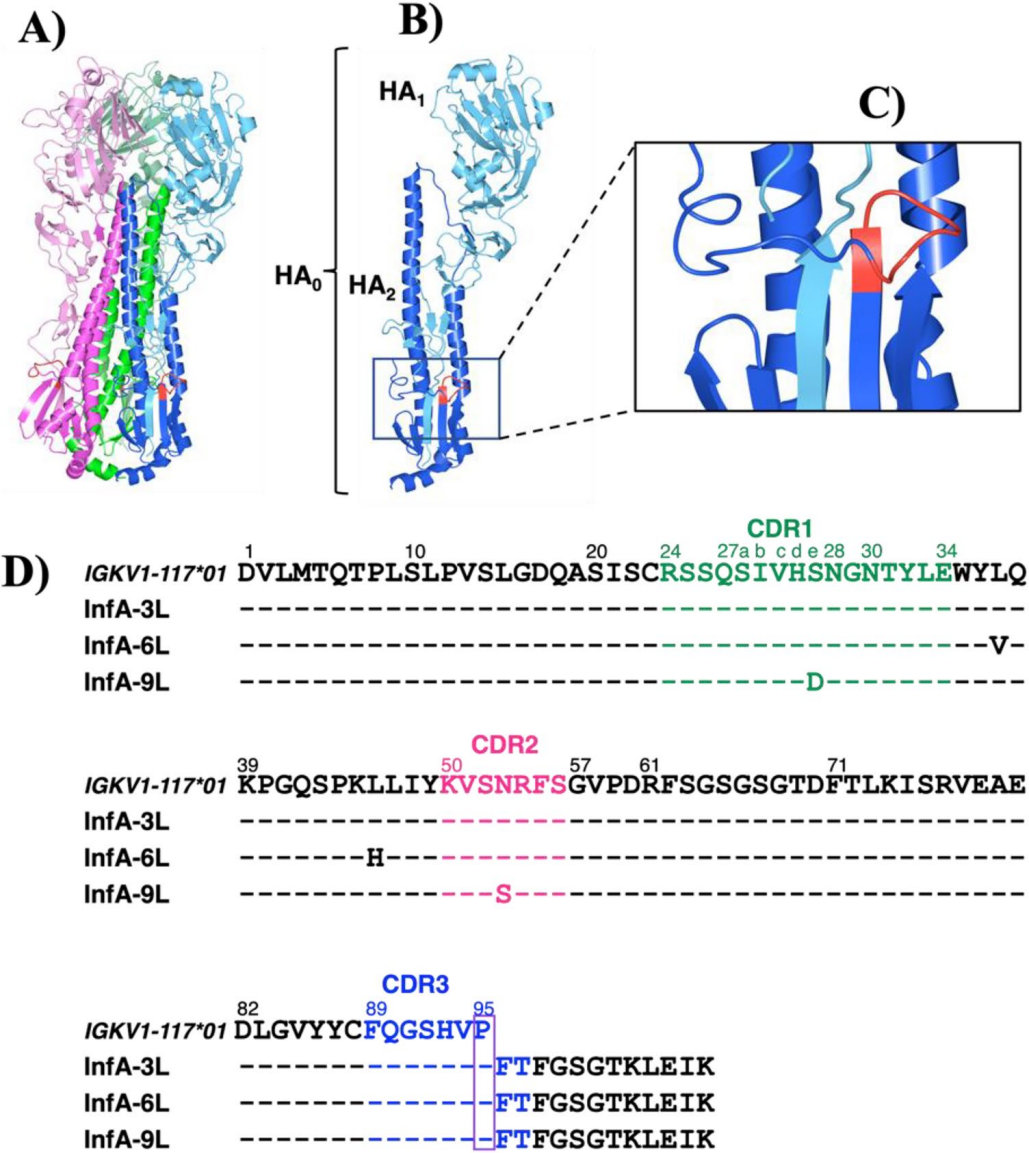
The structure of HA, InfA-peptide, and aa sequences of the antibody light chains. The GMVDGWYG peptide location is indicated in red. (**A**) Trimeric form of hemagglutinin (HA) from influenza A virus. (PDB: 4LXV). HA_1_ domain (light color); HA_2_ domain (dark color). (**B**) Monomeric form of the HA molecule. HA_1_, light blue; HA_2_, dark blue. (**C**) Enlarged view. The GMVDGWYG peptide (red: InfA peptide) is not buried inside the HA molecule. (**D**) Comparing the amino acid sequences of the antibody light chains. InfA-3 light chain (InfA-3L) has a sequence consistent with that of the germline gene (IGKV1-117*01, indicating that no somatic mutations occurred. In InfA-6L, two amino acids are mutated. The first was at the 37th position (Leu to Val). The other was at the 46th position (Leu to His). In InfA-9L, these two positions were mutated. One was at the 27eth position (Ser to Asp). The other was at the 53rd position (Asn to Ser).

Eighteen subtypes of hemagglutinin molecule (H1-H18) exist in the influenza A virus. The representative subtypes of the aa sequences of the HA_2_ domain of the hemagglutinin molecules of H1 (Spanish flu), H2 (Asian Flu), H3 (Hong Kong Flu), and H5 (Avian Flu) are presented in Supplementary Fig. S1. The 3D structure of the hemagglutinin molecule (HA) is shown in Fig. 1A–C, where Fig. 1A shows the trimeric form of HA and Fig. 1B shows the monomeric form. In HA subtypes, several highly conserved regions exist in the sequence. One of them is a GMVDGWYG sequence (designated as the InfA-peptide), which is located at aa 387–394 of the HA molecule (Supplementary Fig. S1). The GMVDGWYG position is indicated in red in Fig. 1A–C, where the entire hemagglutinin molecule (HA_0_) is shown (HA_1_ domain, light blue; HA_2_ domain, dark blue). Figure 1C shows that the peptide is a loop that is not buried inside the HA molecule.

Herein, three types of mice mAbs, InfA-3, -6, and -9, raised against the conserved region of the peptide GMVDGWYG, were used to investigate whether these mice-type mAb light chains could be converted to the corresponding catalytic antibody light chain. Conversion to a catalytic antibody (that is enzymatization) can be accomplished by deleting Pro95, as previously stated^26,27^. These three antibodies belong to the identical germline gene IGKV1-117*01. The sequences of the light chains are shown in Fig. 1D. The V gene of the InfA-3 light chain (InfA-3L) is consistent with the germline gene, indicating that no somatic mutations occurred during maturation. Contrastingly, two somatic mutations occurred at the 37th and 46th positions for InfA-6L, at the 27eth and 53rd positions for InfA-9L. Thus, examining the influence of somatic mutations on the enzymatization to catalytic antibodies is possible.

### Preparation and SDS–PAGE analysis

**Figure 2:**
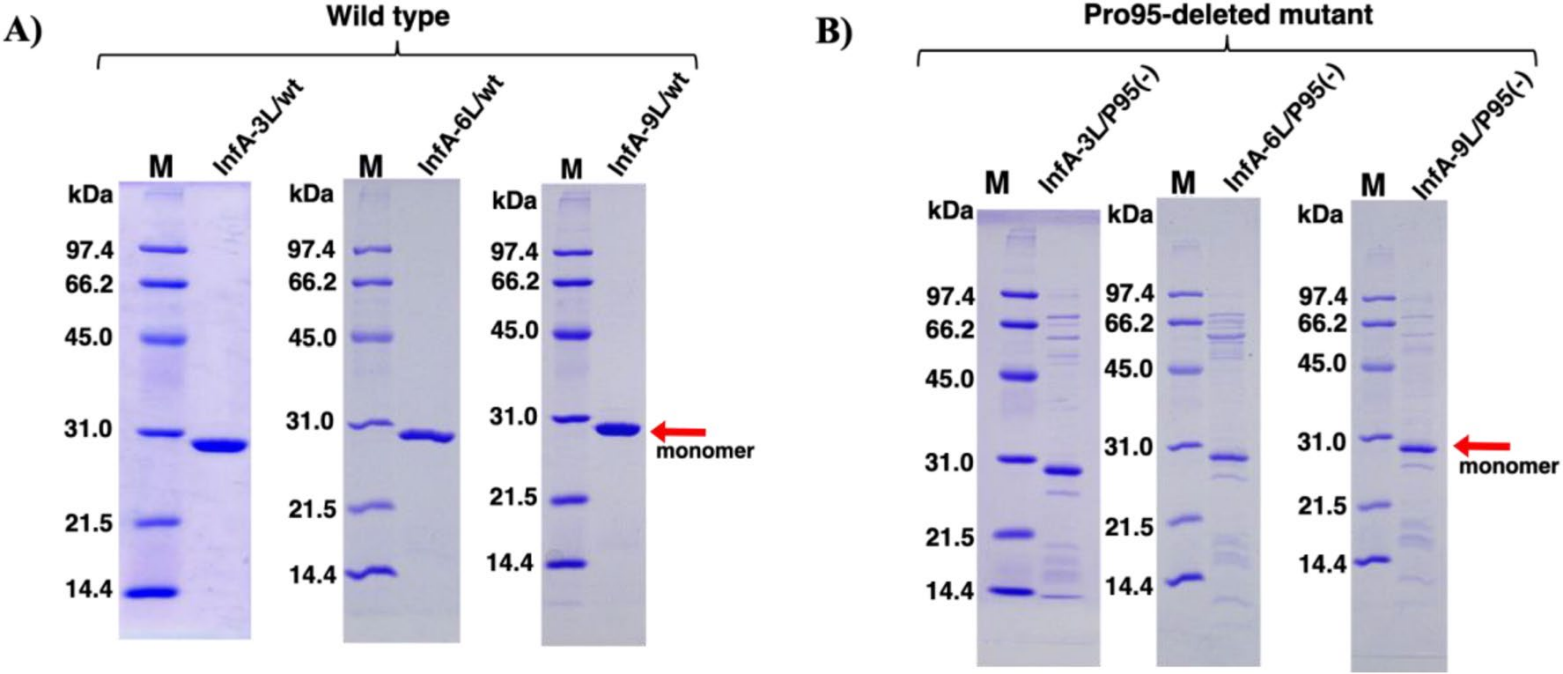
SDS–PAGE analysis. SDS–PAGE analysis performed under reduced conditions is shown with Coomassie brilliant blue staining. (Original gels are presented in supplementary information). M, marker. (**A**) InfA-3L/wt, InfA-6L/wt, and InfA-9L/wt. For all wild-type light chains, the band observed at approximately 29 kDa corresponded to the monomer. (**B**) InfA-3L/P95(−), InfA-6L/P95(−), and InfA-9L/P95(−). In all Pro95(−) mutants, a band of a similar size (approximately 28 kDa) was detected. Although several faint bands, except for the monomer, were detected in the Pro-deleted mutants, these did not affect the FRET-HA peptide cleavage.

The InfA-3L, InfA-6L, and InfA-9L wild types (wts) were prepared as per the procedures described in the “Methods” section. These were referred to as InfA-3L/wt, InfA-6L/wt, and InfA-9L/wt, respectively. The mutants without Pro95 of CDR-3 (InfA-3L/P95(−), InfA-6L/P95(−), and InfA-9L/P95(−)) were produced by genetically deleting the Pro95 residue in the light chain. These six proteins were expressed in the *E. coli* system cultured at 18 °C, recovered by centrifugation from the supernatant, and submitted to purification. The purity and molecular form of the three purified light chains and three mutants were examined using SDS–PAGE with Coomassie brilliant blue (CBB) staining (Fig. 2A,B), where all data were collected under reduced conditions. Figure 2A shows the results for the wild-type InfA-3L, InfA-6L, and InfA-9L. Figure 2B displays those for the three mutants of InfA-3L/P95(−), InfA-6L/P95(−), and InfA-9L/P95(−). In all cases, the band observed at approximately 28–29 kDa corresponded to the monomeric form of each light chain.

### Peptidase activity tests

#### Designing the FRET-HA peptide

**Figure 3:**
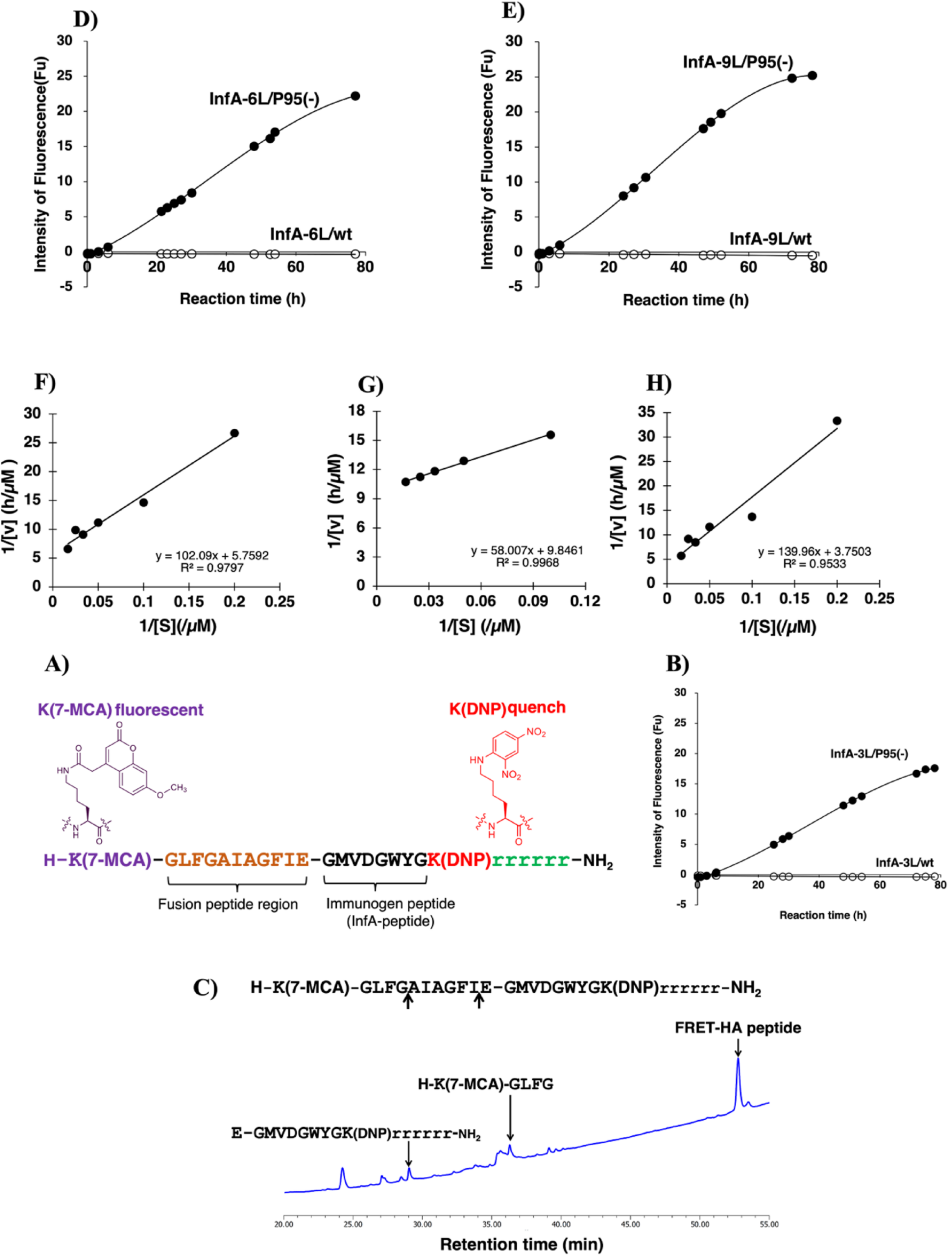
FRET-HA peptide and cleavage reaction. (**A**) The chemical structure of the FRET-HA peptide. The peptide GLFGAIAGFIE (aa 372–382; part of the fusion peptide) was added before GMVDGWYG (aa 387–394; immunogen peptide) owing to GMVDGWYG being hydrophobic. The fluorescent reagent 7-MCA and quenching reagent DNP were bound to Lys. These reagents were introduced into the N- or C-terminus of the GLFGAIAGFIE-GMVDGWYG peptide. Additionally, six D-arginine residues (D-Arg)_6_ [rrrrrr] were added to enhance peptide hydrophilicity. (**B**) Time course of the cleavage reaction of InfA-3L/wt and InfA-3L/P95(−). FRET-HA peptide, 25 μM; InfA-3L/wt (open circle), 5 μM; InfA-3L/P95(−) (closed circle), 5 μM. InfA-3L/wt did not exhibit any catalytic activity for cleaving the FRET-HA peptide. Contrastingly, InfA-3L/P95(−) clearly decomposed the FRET-HA peptide in a time-dependent manner. (**C**) Cleaved peptide bond. The reaction products obtained in the above experiment were analyzed using HPLC and MS. Some peaks were observed, as shown in the chromatogram. Two of these peaks were detected. The first was obtained from E-GMVDGWYGK(DNP)rrrrrr-NH_2_ and the other was H–K(7-MCA)-GLFG. InfA-3L/P95(−) cleaved the peptide bond between G–A and I–E. (**D**) The time course of the cleavage reaction of InfA-6L/wt and InfA-3L/P95(−). FRET-HA peptide, 25 μM; InfA-6L/wt (open circle), 5 μM; InfA-6L/P95(−) (closed circle), 5 μM. InfA-6L/wt did not exhibit any catalytic activity for cleaving the FRET-HA peptide. Contrastingly, InfA-6L/P95(−) clearly decomposed the FRET-HA peptide in a time-dependent manner. (**E**) The time course of the cleavage reaction of InfA-9L/wt and InfA-9L/P95(−). FRET-HA peptide, 25 μM; InfA-9L/wt (open circle), 5 μM; InfA-9L/P95(−) (closed circle), 5 μM. InfA-9L/wt did not exhibit any catalytic activity for cleaving the FRET-HA peptide. Contrastingly, InfA-9L/P95(−) clearly decomposed the FRET-HA peptide in a time-dependent manner. (**F**–**H**) represent the results of the kinetic analysis under the following conditions: The concentration of mutant such as InfA-3L/P95(−), InfA-6L/P95(−), and InfA-9L/P95(−) was fixed at 5 μM and that of the FRET-HA peptide varied from 5 to 600 μM at 37 °C. [S], FRET-HA peptide; [v], initial rate. (**F**) InfA-3L/P95(−). The Lineweaver–Burk plot demonstrated that the cleavage reaction by the InfA-3L/P95(−) mutant fits the Michaelis–Menten kinetics equation, indicating that the reaction is enzymatic. (**G**) InfA-6L/P95(−). InfA-6L/P95(−) exhibited a linear relationship between 1/[v] vs. 1/[S], indicating that degradation by the mutant obeyed the Michaelis–Menten kinetics. (**H**) InfA-9L/P95(−). The degradation reaction obeyed Michaelis–Menten kinetics, indicating that the degradation was enzymatic.

Three mAbs (InfA-3, InfA-6, and InfA-9) were produced by immunizing a conservative region peptide (GMVDGWYG: HA_2_ (aa 387–394)) conjugated with bovine serum albumin (BSA)^29^. In order to investigate the catalytic feature to degrade hemagglutinin molecule (HA) of influenza A virus, we synthesized a FRET-HA peptide substrate whose chemical structure is shown in Fig. 3A. Considering the low solubility of the GMVDGWYG peptide in aqueous buffer, the peptide of aa 372–382 (GLFGAIAGFIE: a part of the fusion peptide) of HA_2_ was introduced before the GMVDGWYG sequence. Additionally, six D-arginine residues (D-Arg)_6_ [rrrrrr] were added to enhance peptide hydrophilicity. Finally, the synthesized FRET-HA peptide was confirmed using high-performance liquid chromatography (HPLC) and mass spectrometry (MS) (Supplementary Fig. S2A). The quenching performance of the synthesized FRET-HA peptide was also investigated (Supplementary Fig. S2B). The quenching rate was > 100-fold, which was satisfactory for subsequent experiments.

#### Cleavage reaction of the FRET-HA peptide

Using the FRET-HA peptide, catalytic activity (peptidase activity) was examined for all mAbs, light chains, and Pro95-deleted light chains. For InfA-3, -6, and -9 mAbs, no cleavage activity was exhibited (see Supplementary Fig. S3).

The time courses of the cleavage reaction of InfA-3L/wt and InfA-3L/P95(−) mutant are presented in Fig. 3B. Although InfA-3L did not cleave the FRET-HA peptide, InfA-3L/P95(−) mutant could cleave the FRET-HA peptide in a reaction-time dependent manner. In the cleavage of the FRET-HA peptide by InfA-3L/P95(−), the scissile peptide bond was investigated using HPLC and MS. Several fragments were observed (Fig. 3C). The peak at 29.1 min was identified to be E-GMVDGWYGK(DNP)rrrrrr-NH_2_ and that at 36.5 min was identified to be H–K(7-MCA)-GLFG, suggesting that the peptide bond between G–A and I–E was cleaved by the InfA-3L/P95(−) mutant.

The time courses of the FRET-HA peptide degradation by InfA-6L/wt and InfA-6L/P95(−) are presented in Fig. 3D. In this case, the same degradation time courses were observed as those obtained in Fig. 3B. Additionally, similar results were obtained for InfA-9/wt and InfA-9L/P95(−) (Fig. 3E).

Kinetic studies were performed for the three catalytic antibody light chains, InfA-3L/P95(−), InfA-6L/P95(−), and InfA-9L/P95(−), by varying the FRET-HA peptide concentration while keeping the concentration of the catalytic antibody light chain constant at 5 μM.

Figure 3F–H show the Lineweaver–Burk plots for the cleavage of the FRET-HA peptide by InfA-3L/P95(−), InfA-6L/P95(−), and InfA-9L/P95(−), respectively. The reaction catalyzed by InfA-3L/P95(−) fits the Michaelis–Menten equation, suggesting that degradation of the substrate by the Pro95-deleted mutant is enzymatic (Fig. 3F). The dissociation constant (Km) was found to be 17.7 × 10^−6^ M. The catalytic reaction constant (kcat) was 5.8 × 10^−4^ min^−1^. The catalytic efficiency (kcat/Km) was 32.6 M^−1^ min^−1^.These values are comparable to those previously reported. Paul et al.^4^ found that the anti-vasoactive intestinal peptide (VIP) antibody light chain cleaved the substrate VIP with Km = 0.2 × 10^−6^ M and kcat = 1.1 × 10^−2^ min^−1^. Durova et al.^21^ obtained the values of Km = 11.5 × 10^−6^ M and kcat = 6.8 × 10^−3^ min^−1^ for Pro-Phe-Arg-MCA substrate using the antibody light chain (L12). Table 1 summarizes the kinetic values of kcat and Km for three mutants of InfA-3L/P95(−), InfA-6L/P95(−), and InfA-9L/P95(−). The kcat and Km values were not considerably different for each light chain. Somatic mutations occurring in InfA-6L/wt and InfA-9L/wt did not affect the cleavage ability of the antigen. Nevertheless, all mice antibody light chains achieved enzymatic function by deleting the CDR-3 Pro95 residue.

**Table 1:** Kinetic parameters for the cleavage reaction of the FRET-HA peptide.

Clone name	K_m_ (M)	k_cat_ (min^−1^)	k_cat_/K_m_ (min^-1^ M^-1^)
InfA-3L/P95(−)	17.7 × 10^−6^	5.8 × 10^−4^	32.6
InfA-6L/P95(−)	5.9 × 10^−6^	3.4 × 10^−4^	57.4
InfA-9L/P95(−)	37.3 × 10^−6^	8.9 × 10^−4^	23.8

### Immunological features

#### Enzyme-linked immunosorbent assays (ELISAs)

ELISA experiments were performed using the antigenic peptide (CGMVDGWYG) as the coated antigen to investigate the correlation between the Michaelis constant (Km) and the affinity constant (K) for each sample used in this study.

**Figure 4:**
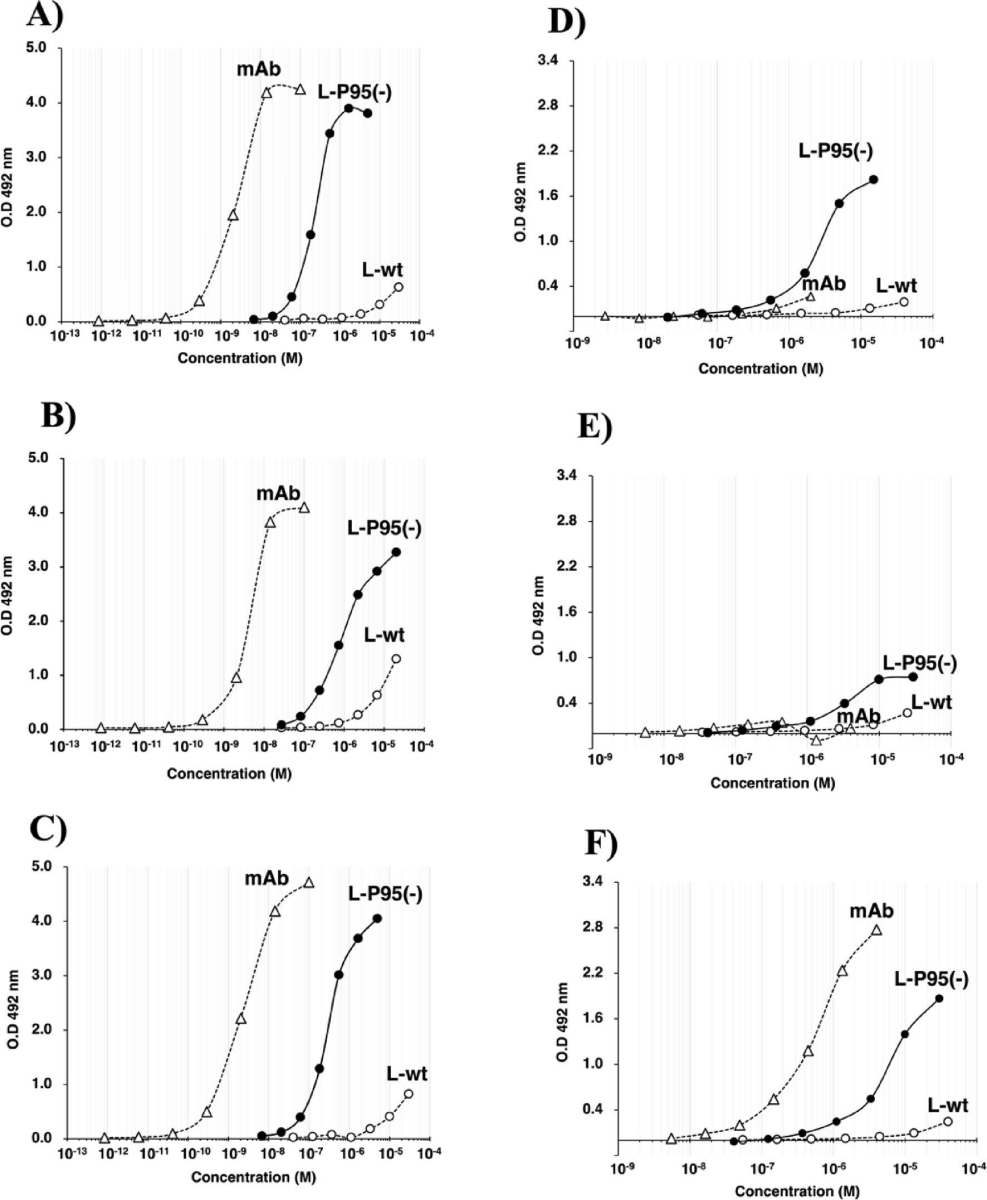
ELISAs. *For the peptide antigen*: BSA-CGMVDGWYG antigen (5 μg/mL) was coated on the immunoplate. After InfA mAb, InfA-L/wt, or InfA-L/Pro95(−) reacted with the coated antigen, POD-labeled goat affinity-purified antibody to mouse IgG (Fab) was added to the wells, followed by color development with o-phenylenediamine. (**A**) ELISA for InfA-3 mAb, InfA-3L/wt, or InfA-3L/P95(−). mAb and the P95(−) mutant exhibited a typical sigmoid curve, and the affinity constants (K) were 4.15 × 10^8^ /M and 4.22 × 10^6^ /M, respectively. The immunoreactivity of InfA-3L/wt was low, and the K value was < 8.25 × 10^4^ /M. (**B**) ELISA for InfA-6 mAb, InfA-6L/wt, or InfA-6L/P95(−), The affinity constants (K) for mAb and P95(−) mutant were 2.42 × 10^8^ /M and 1.19 × 10^6^ /M, respectively, and the K value for InfA-6L/wt was < 1.30 × 10^4^ /M. (**C**) ELISA for InfA-9 mAb, InfA-9L/wt, or InfA-9L/P95(−). The affinity constants (K) for mAb and P95(−) mutant were 4.08 × 10^8^ /M and 3.43 × 10^6^ /M, respectively, and the K value for InfA-9L/wt was < 8.73 × 10^4^ /M. *For the recombinant H1N1*: Recombinant H1N1 (rH1N1; 5 μg/mL) was coated on the immunoplate. The procedure was the same as that described above. (**D**) ELISA for InfA-3 mAb, InfA-3L/wt, or InfA-3L/P95(−). Neither InfA-3 mAb nor InfA-3L/wt reacted with rH1N1. InfA-3L/P95(−) exhibited a typical sigmoid curve, from which the affinity constant was estimated to be 4.11 × 10^5^ /M. (**E**) ELISA for InfA-6 mAb, InfA-6L/wt, or InfA-6L/P95(−). Only InfA-6L/P95(−) exhibited a sigmoid curve. The K value was 4.07 × 10^5^ /M. InfA-6 mAb did not react with rH1N1. InfA-6L/wt showed a weak reaction to rH1N1. However, an accurate K value was not estimated. (**F**) ELISA for InfA-9 mAb, InfA-9L/wt, or InfA-9L/P95(−). InfA-9 mAb and InfA-9L/Pro95(−) exhibited sigmoid curves, with K values of approximately 1.82 × 10^6^ and approximately 1.98 × 10^5^ /M, respectively.

InfA-3 mAb exhibited a clear sigmoid curve (Fig. 4A). The affinity constant (K) was estimated from the curve as 4.15 × 10^8^ /M, which was of the same order as that obtained via isothermal titration calorimetry, as previously reported^29^. (The derivation method of K value from ELISA experiment is explained in Supplementary Fig. S4, where the case of InfA-6 mAb is figured.). Contrastingly, the K value of InfA-3L/wt was < 8.25 × 10^4^ /M (in this case, the accurate measurement was not possible, as the value was too small to be evaluated). For InfA-3L/P95(−), the affinity constant (K) was 4.22 × 10^6^/M. Interestingly, the K value was enhanced by a factor of approximately 50-fold compared with that of InfA-3L/wt. Similar results were obtained for the InfA-6 (InfA-6 mAb, InfA-6L/wt, and InfA-3L/P95(−); Fig. 4B)) and InfA-9 series (Fig. 4C). These values are summarized in Table 2. Conclusively, the affinity constant was in the order of approximately 10^8^ /M for mAb, approximately 10^6^ /M for L/P95(−), and < 10^4^ /M for L/wt.

**Table 2:** Affinity constants for the antigenic peptide.

Clone name	Affinity constant (/M)	Clone Name	Affinity constant (/M)	Clone Name	Affinity constant (/M)
InfA-3 mAb	4.15 × 10^8^	Infa-6 mAb	2.42 × 10^8^	Infa-9 mAb	4.08 × 10^8^
InfA-3L/wt	≤ 8.25 × 10^4^	Infa-6L/wt	≤ 1.30 × 10^4^	Infa-9L/wt	≤ 8.73 × 10^4^
Infa-3L/Pro95(−)	4.22 × 10^6^	InfA-6L/Pro95(−)_	1.19 × 10^6^	InfA-9L/Pro95(−)	3.43 × 10^6^

Next, ELISA experiments using recombinant H1N1 (rH1N1) as the coated antigen were performed for the InfA-3 (mAb, light chain/wt, and mutant), InfA-6, and InfA-9 series. The results are shown in Figs. 4D–F. Although InfA-3 mAb and InfA-6 mAb hardly reacted with rH1N1, InfA-9 mAb did. Neither InfA-3 mAb nor InfA-3L/wt reacted with rH1N1; however, InfA-3L/P95(−) reacted. Similarly, InfA-6 mAb and InfA-6L/wt did not react with rH1N1, whereas InfA-6L/P95(−) did. The K value of InfA-3L/P95(−) was 4.11 × 10^5^ /M and that of InfA-6L/P95(−) was 4.07 × 10^5^ /M. For InfA-9L/P95(−), the K value was 1.98 × 10^5^ /M. The three mutants exhibited similar affinity constants in the order of approximately 10^5^ /M. The relationship between Km and K obtained above will be discussed in a later section.

### Analysis of catalytic site

**Figure 5:**
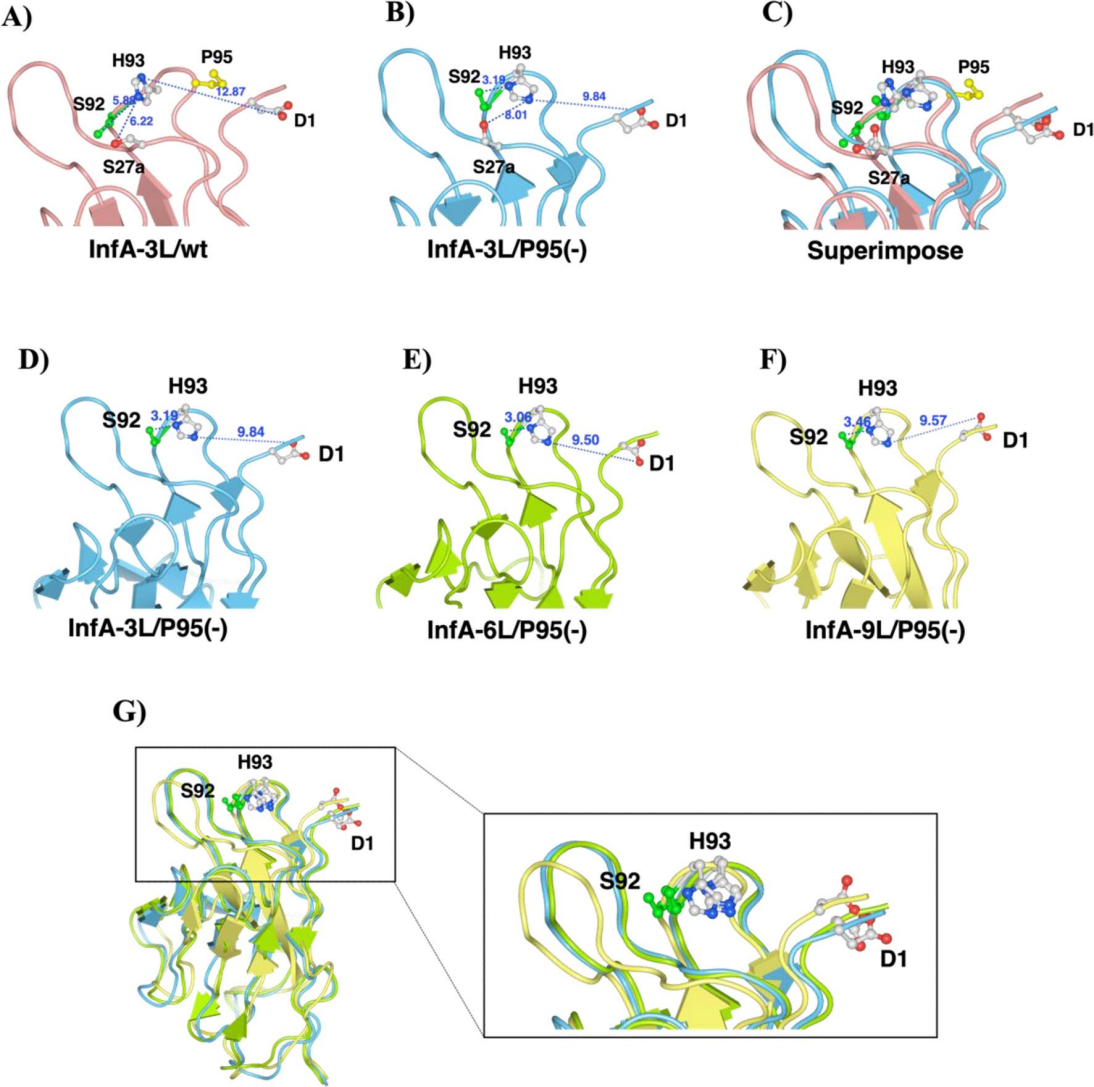
Analysis of catalytic site. Red ball, oxygen; blue ball, nitrogen; green ball and stick, Ser92 residue. (**A**) InfA-3L/wt, the main chain is light red. (**B**) InfA-3L/P95(−), the main chain is light blue. (**C**) Superimposed view of (**A**) and (**B**). The conformation of His93 changed from vertical in wt to horizontal in the P95(−) mutant. The distance between Ser92(O) and His93(N) was 5.88 Å in InfA-3L/wt (A) and 3.19 Å in InfA-3L/P95(−) (**B**). The distance of Asp1-His93(N) of InfA-3L/wt was 12.87 Å and that of InfA-3L/P95(−) was 9.84 Å, which became shorter than that of InfA-3L/wt by 3.03 Å. Asp1, Ser92, and His93 residues were the most possible catalytic sites. (**D**) InfA-3L/P95(−), main chain is represented in cyan. Ser92(O)-His(N) = 3.19 Å and Asp1(O)-His(N) = 9.84 Å. (**E**) InfA-6L/P95(−), main chain is represented in light green. Ser92(O)-His(N) = 3.06 Å and Asp1(O)-His(N) = 9.50 Å. (**F**) InfA-9L/P95(−), main chain is represented in yellow. Ser92(O)-His(N) = 3.46 Å, Asp1(O)-His(N) = 9.57 Å. (**G**) Superimposed view of (**D**–**F**). The distances between Ser92(O)-His(N) take preferable position in all mutants of InfA-3L/P95(−), InfA-6L/P95(−), and InfA-9L/P95(−). The conformations and the positions of Asp1, Ser92, and His93 did not change in any of the three Pro95-deleted mutants.

Based on the studies on the catalytic features of antibodies, the aa residues Asp, Ser, and His are considered to form a catalytic triad-like structure in many cases^4,9,10,19,20,25–27^. InfA-3, -6, and -9 light chains have characteristic residues such as Asp1, Ser27a (or Ser92), and His93, which can potentially generate a catalytic triad. However, the light chains of the wild-type/wt did not exhibit any catalytic activity. Figure 5A–C show the structural models of InfA-3L/wt, InfA-3L/P95(−), and their superimposed views, respectively. The figures clearly represent the changes in the His93 conformation from vertical in the wt to horizontal in the P95(−) mutant.

Regarding the relative positions between His93(N) and Ser92(O), the distance of two residues was changed from 5.88 Å of InfA-3L/wt to 3.19 Å of InfA-3L/P95(−). (Fig. 5A,B). In other words, Pro95 deletion resulted in the shortening of the distance by 2.69 Å. On the other hand, the distance between Ser27a(O) and His93(N) was 8.01 Å in InfA-3L/P95(−) and 6.22 in InfA-3L/wt. Pro95 deletion lengthens their distance, which implies that Ser27a is not involved in generating a catalytic triad in this case. Regarding the relation between Asp1 and His93, the distance of Asp1(O)-His93(N) of InfA-3L/P95(−) is 9.84 Å, which is shorter than the distance of the InfA-3L/wt (11.87 Å) by 3.03 Å. By deleting Pro95, the residues Asp1 and Ser92 came closer to the His93 residue, resulting in the acquisition of a preferable position. The same phenomena were observed for InfA-6L/wt vs InfA-6L/P95(−) and InfA-9L/wt vs InfA-9L/P95(−).

Figure 5D–F show the InfA-3L/P95(−), InfA-6L/P95(−), and InfA-9L/P95(−) structures, respectively. Table 3 summarizes the distances (Å) between Asp1(O)-His93(N) and His93(N)-Ser92(O). For the cases of Pro95-deleted mutants, similar tendencies were observed, in which both Asp1(O)-His93(N) and His93(N)-Ser92(O) distances were shortened by Pro95 residue deletion. The conformations and positions of Asp1, Ser92, and His93 remained unchanged (Fig. 5G). It appears that Pro95 deletion in the CDR-3 contributes to the formation of a preferable catalytic site that cleaves the antigen.

**Table 3:** Distances between the important amino acid residues.

	Asp1(O)-His93(N) (Å)	His93(N)-Ser92(O) (Å)
InfA-3L/wt	12.87	5.88
InfA-3L/P95(−)	9.84	3.19
InfA-6L/wt	11.68	5.4
InfA-6L/P95(−)	9.5	3.06
InfA-9L/wt	11.41	4.07
InfA-9L/P95(−)	9.57	3.46

### In vitro assays

The prepared samples were subjected to in vitro assays to examine their neutralization ability against the influenza A virus H1N1 (A/Hiroshima/37/2001) using the Madin–Darby canine kidney cells (MDCK). Herein, the samples were first incubated with 2,000 pfu/mL influenza virus H1N1 for 24 or 48 h at 25 °C and subsequently, the mixture was inoculated into the MDCK cell monolayer (see “Methods”) to evaluate infectivity via counting the number of plaques on the plates.

**Figure 6:**
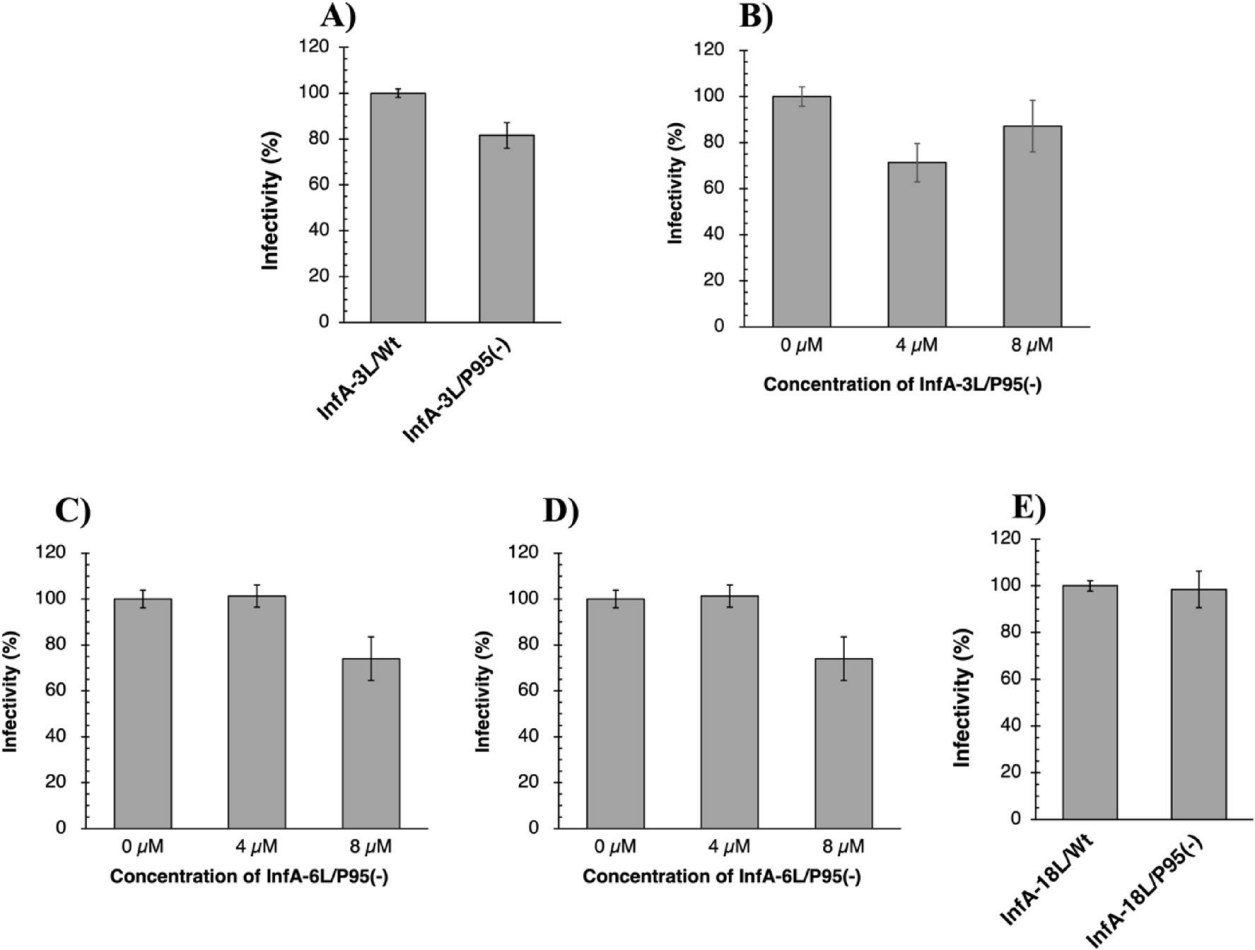
In vitro assays. Reaction conditions included Madin–Darby canine kidney (MDCK) cells (6.0 × 10^4^ cells/cm^2^), influenza A virus (H1N1; 2,000 PFU/mL), and incubation (48 h at 25 °C). (**A**) InfA-3L/wt and InfA-3L/P95(−). InfA-3L/wt (12 μM; n = 2) exhibited no neutralization effect; however, InfA-3L/P95(−) (12 μM; n = 2) suppressed the virus infection by a factor of approximately 20%. (**B**) The concentration dependency of InfA-3L/P95(−). InfA-3L/P95(−) reduced the infectivity at 4 and 8 μM. The neutralizing efficacy was approximately 30% at 4 μM. (n = 2). (**C**) The concentration dependency of InfA-6L/P95(−). InfA-6L/P95(−) infectivity was reduced in a concentration-dependent manner. The effective concentration was observed at 8 μM. The neutralizing efficacy was approximately 25%. (n = 4). (**D**) The concentration dependency of InfA-9L/P95(−). InfA-9L/P95(−) exhibited a neutralization effect in a concentration-dependent manner. The most effective concentration was 8 μM. The neutralizing efficacy was approximately 20%. (n = 2). (**E**) InfA-18L/wt and InfA-18L/P95(−). Both InfA-18L/wt (n = 4) and InfA-18L/P95(−) (n = 4) hardly showed the neutralization effect for the virus infection.

First, we examined the neutralization ability of InfA-3L/wt and InfA-3L/P95(−), and the results are indicated in Fig. 6A. Although InfA-3L/wt exhibited no neutralization effect, InfA-3L/P95(−) exhibited approximately 20% suppression of viral infectivity at 48 h of incubation. In a previous study^11^, the human antibody light chain 23D4 (2 μM) exhibited approximately 20% and approximately 35% suppression of viral infectivity after 24 and 48 h of incubation, respectively. Therefore, the InfA-3L/P95(−) concentrations varied from 0 to 8 μg/mL as the next experiment. The results are shown in Fig. 6B. At 4 and 8 μM, InfA-3L/P95(−) reduced the infectivity. The neutralization effect was approximately 30% at 4 μM. Similar experiments were performed for the InfA-6L/P95(−) and InfA-9L/P95(−) light chains. Figure 6C,D show the results for InfA-6L/P95(−) and InfA-9L/P95(−), respectively. For InfA-6L/P95(−), a neutralization effect of approximately 25% was observed at 8 μM. The InfA-9L/P95(−) light chain affected the infection rate by approximately 20% at 8 μM. In this in vitro assay, all Pro95-deleted mutants exhibited the ability to suppress the influenza virus infection.

## Discussion

MAbs are mainly classified as human, mouse, or chimeric. As previously reported, we succeeded in converting human mAbs into catalytic antibodies (i.e., enzymatization). Herein, we investigated whether the enzymatization of mouse mAb to its catalytic antibody is possible.

We used three mice mAbs, InfA-3, InfA-6, and InfA-9, which were produced by immunizing Balb/c mice with the highly conserved sequence peptide GMVDGWYG (aa 387–394) conjugated with BSA^29^. In the mouse antibody light chain, kappa VL germline genes, such as IGKV1-117*01, IGKV1-88*01, IGKV1-135*01, IGKV1-110*01, IGKV1-122*01, and IGKV1-133*01, have a catalytic triad composed of three amino acid residues, Asp, Ser, and His^30^. InfA-3L, InfA-6L, and InfA-9L belong to the IGKV1-117*01 germline gene family. Therefore, the three light chains inherently contain the typical amino acids Asp1, Ser27a (or Ser92), and His93, which have the potential to construct a catalytic triad. However, the light chains and mAbs do not exhibit catalytic (peptidase) activity.

Contrastingly, Pro95-deleted mutants (InfA-3L/P95(−), InfA-6L/P95(−), and InfA-9L/P95(−)) obviously exhibited peptidase activity to cleave the FRET-HA peptide regardless of the occurrence of the somatic mutation (Fig. 3B,D,E). Taking these into account, we must consider why and how the Pro95-deleted light chain obtains the catalytic function and we must determine the cleavage ability of the peptide.

Several studies on the catalytic sites have been reported so far. A catalytic triad composed of His, Ser, and Asp (or catalytic dyad) is crucial in the catalytic cleavage reaction in terms of a serine protease-like mechanism. These catalytic sites have been confirmed using site-directed mutagenesis studies^31,32^, X-ray crystallography^33^, mass spectrometry^33^, and other methods^4,26,34^. Molecular modeling analysis in the present study revealed that the distances between Asp1(O)-His93(N) and His93(N)-Ser92(O) are shortened by several angstroms by deleting Pro95 in all cases of InfA-3L/P95(−), InfA-6L/P95(−), and InfA-9L/P95(−). Three amino acids (Asp, Ser, and His) came closer to the appropriate positions (Table 3) and subsequently generated the active site for the hydrolysis of the antigenic peptides, which enables the catalytic function acquisition. Thus, a catalytic triad composed of Asp1, Ser92, and His93 is considered to function in the Pro95-deleted mutants.

Contrastingly, the affinity constants between the wt and mutant (Pro95(−)) were largely different, as observed in the ELISA experiments. In the mutants, the affinity constant was enhanced by a factor of approximately two orders of magnitude (approximately 100-fold) compared to that of the wild-type. Proline is a well-known, structurally rigid amino acid residue. Therefore, the light chain with the Pro95 residue (wt) had lower flexibility than the mutant without the Pro95 residue. As shown in Table 2, the K values of InfA-3L/wt, -6L/wt, and -9L/wt are lower than those of InfA-3L/Pro95(−), -6L/Pro95(−), and -9L/Pro95(−), respectively. Roughly estimated, the relationship appears to be proportional. Namely, the higher the K value is, the greater the flexibility becomes, though quantifying flexibility is difficult. Flexibility enhancement is considered to lead to a strong interaction with the antigenic molecule through an event such as induced fitting. Overall, Pro95 deletion contributes to the catalytic function and enhances the recognition ability.

A comparison of the affinity constant (K) obtained using ELISA with the Michaelis–Menten constant (Km) obtained via kinetic studies can provide information on the recognition and catalytic sites. The K value represents the binding affinity of the antigen to the antigen-recognition site of the antibody. Contrastingly, the 1/Km value corresponds to the binding affinity of the substrate to the catalytic site. The K and I/Km values of InfA-3L/P95(−) were 4.22 × 10^6^ /M and 5.6 × 10^4^ /M, respectively and these values differed by seventy-five-fold. As previously reported, in the case of the anti-gp41 antibody light chain (against the HIV envelope protein)^35^, the antigen recognition and catalytic sites were the same, because the values (K vs. 1/Km) were in the same order (~ 10^6^/M). On the contrary, for H34 antibody light chain, these values differed by two orders of magnitude^36^. Hence, it is concluded that the antigen recognition and catalytic sites were located in different parts in the case of InfA-3L/P95(−). Regarding InfA-6L/P95(−) and InfA-9L/P95(−), the differences of K and 1/Km values were 7- and 127-fold, respectively. Taking these results into account, the antigen recognition site and active site in Pro95 deleted mutants are not identical but different.

The InfA-3L/P95(−) mutant possessed the ability to suppress the influenza A virus (H1N1 virus) infectivity, although mAb and InfA-3L/wt did not show this effect (Fig. 6A). The approximately 30% inhibitory effect observed in the InfA-3L/P95(−) mutant was the same as that obtained using the human catalytic antibody light chain 23D4, which was effective in preventing viral infection in in vivo assays^11^. A similar infection suppression was observed for InfA-6L/P95(−) and InfA-9L/P95(−). Notably, the results of the in vitro assays agree well with those obtained in the catalytic activity assays, indicating that the enzymatization of the mouse mAb is possible and that new functions have emerged.

Not all Pro95-deleted mutants showed a neutralizing effect. As previously mentioned^18^, the intrinsic presence of certain types of catalytic sites such as triads^3,9,23^ and dyads^34,37^, among others^11,38^, is necessary for acquiring a catalytic function capable of cleaving the antigen molecule. In this study, we performed a control experiment using InfA-18 mAbs, which were simultaneously produced with InfA-3, -6, and -9 mAbs. The light chain of InfA-18 mAb (InfA-18L/wt) belongs to the germline gene IGKV6-23*01, which has no catalytic triad in its structure. InfA-18L/wt and InfA-18L/P95(−) did not exhibit catalytic activity to cleave the HA peptide and the neutralization effect on the influenza virus (Fig. 6E).

In conclusion, the mouse mAb light chain can be enzymatized by deleting the Pro95 residue in the CDR-3. Consequently, the Pro95-deleted catalytic antibody could cleave the peptide antigen and exert a suppressive function against the influenza virus infection.

## Methods

### Reagents

Chemical reagents such as Tris, glycine, CuCl_2_·2H_2_O, KCl, Na_2_HPO_4_·12H_2_O, NaCl, KH_2_PO_4_, EDTA·2Na, and IPTG were purchased from Wako Pure Chemical Industries Ltd., Osaka, Japan (Guaranteed Reagent). The synthetic substrates peptidyl-pNA and Arg-pNA were purchased from the Peptides Institute, Inc. (Osaka, Japan). Tryptone and yeast extracts were purchased from Becton–Dickinson and Company, NJ, USA. Commercially available recombinant hemagglutinin molecules (rH1N1 and rH3N2) were purchased from Sino Biochemical Inc. (Product Numbers: 11085-V08B (H1N1); 11715-V08B (H3N2), Beijing, China).

### FRET substrate synthesis

The FRET-HA peptide (7-MCA-GLFGAIAGFIE-GMVDGWYGK(DNP)rrr-NH_2_) was synthesized on a solid support using the Fmoc/tBu strategy on a rink amide resin as previously reported^39^. Briefly, the Fmoc group was removed with 20% piperidine in dimethylformamide, and chain elongation was achieved by standard HBTU/HOBt chemistry using three equivalents of protected amino acids or 7-MCA. After synthesis completion, the protected peptide resin was treated with a TFA/phenol/H_2_O/thioanisole/1,2-ethanedithiol (82.5:5:5:2.5, v/v/v/v) mixture. The crude material obtained was purified using HPLC. The structures of the FRET peptides were confirmed using MS.

### mAbs

The mAbs against InfA-3, InfA-6, and InfA-9 were obtained by immunizing BALB/c mice with BSA-C-GMVDGWYG. After spleen cell fusion with NS-1 cells, screening was performed using GMVDGWYG-C-human IgG, as previously described^29^. mAbs were obtained by purifying the hybridoma culture medium.

#### DNA fragment amplification encoding the light chains

The genes of InfA-3L/wt, InfA-6L/wt, and InfA-9L/wt were obtained using the corresponding cDNA synthesized from hybridomas secreting InfA-3, InfA-6, or InfA-9 mAbs. First, the total RNA was extracted from a hybridoma secreting a mAb, and cDNA was synthesized using reverse transcriptase. Using the cDNA, InfA-3L/wt, InfA-6L/wt, and InfA-9L/wt were prepared as follows:

The cDNA prepared from InfA-3, -6, and -9 mAb-secreting hybridomas was used as the template for amplifying the corresponding light chain gene using a forward primer 5′-CCATGGATGTTTTGATGACCCAAACTC-3′ and a reverse primer 5′-CTCGAGACACTCATTCCTGTTGAAGCTCT-3′ for all cases, as the amino acid sequences that are the neighbors of Pro95 are identical. The PCR reaction occurred under the following incubation conditions: 60 s at 98 °C, 25 cycles of 10 s at 98 °C for denaturation, 30 s at 66–70 °C for annealing, and 40 s at 72 °C for extension. Finally, the extension was performed for 5 min at 72 °C. Phusion (New England Bio Labs, M0530S, Lot 10,084,002, MA) was used for PCR. The amplified DNA fragment was inserted into the pCR4Blunt-TOPO vector (Invitrogen, Zero Blunt TOPO PCR Cloning Kit) and transformed into DH5α (TOYOBO). The plasmid pCR4Blunt-TOPO was digested with *Nco* I and *Xho* I (New England BioLab) and inserted into the pET20b (+) vector (Novagen, Madison, WI, USA), which was repurified and transformed into BL21(DE3) pLysS for InfA light chain and mutant expression.

#### Site-directed mutagenesis

Site-directed mutagenesis was performed on each clone to remove the Pro95 residue from InfA-3L/wt, InfA-6L/wt, and InfA-9L/wt by performing site-directed mutagenesis on each clone.

#### Constructing the Pro95 deleted mutant

Pro95 deletion from the wt InfA-3L was performed using inverse PCR using the reverse primer 5′-AACATGTGAACCTTGAAAGCAGTAATAAACTC-3′ and forward primer 5′- TTCACGTTCGGCTCGGG-3′. In the experiment, the KOD-Plus-Mutagenesis Kit (TOYOBO, Code SMK-101, Osaka) was used at 68.5 °C annealing temperature. The construct was first transformed into the DH5α cells and finally into the BL21(DE3)pLysS cells for expression. The above primers were used for Pro95 deletion from InfA-6L and InfA-9L, following the procedures described above.

#### Sequencing

All wts and mutants were sequenced with an ABI 3730xl Analyzer (Applied Biosystems, CA, USA) using ABI BigDye™ Terminator v3.1 Cycle Sequencing Kits. GENETIX Ver. 8 (GENETIX, Tokyo, Japan) was used for analyzing the sequence and determining the amino acid sequences.

#### Culture, recovery, and purification

The transformant was grown at 37 °C in 1 L of Luria–Bertani medium containing 100 μg/mL ampicillin to an A600 nm absorbance of 0.6 and subsequently incubated with 0.01 mM IPTG for 20 h at 18 °C. The cells were harvested by centrifugation (3500 × *g*; 4 °C; 10 min) and subsequently resuspended in a 100 mL solution of 250 mM NaCl and 25 mM Tris–HCl at pH 8.0. The cells were lysed via ultrasonication three times for 2 min each in an ice bath, followed by centrifugation (21,475×*g*; 4 °C; 20 min). The supernatant included the recovered expressed mouse light chain.

The supernatant was subjected to Ni–NTA column chromatography (Takara, Otsu, Japan) and equilibrated with 25 mM Tris–HCl (pH 8.0) containing 250 mM NaCl. Elution was performed by increasing the imidazole concentration from 0 and/or 30 to 300 mM. After completing the Ni–NTA column chromatography, an aliquot of a solution of 50 μM CuCl_2_ (2.5 eq for the light chain) was added into the eluent (this is crucial to create a uniform (dimer) structure) based on the calculation that the absorbance of A280 nm of 1.0 in ultraviolet–visible (UV/VIS) spectroscopy was regarded as approximately 1 mg/mL (40 μM light chain). The solution containing the light chain and copper ions was subsequently dialyzed against 50 mM Tris–HCl buffer (pH 8.0) for approximately 20 h. After removing some aggregates via centrifugation (21,475×*g*; 4 °C; 20 min), the solution was concentrated to 2 mg/mL and subjected to cation-exchange chromatography using a column of SP-5PW (TOSOH, Japan) with an NaCl gradient (from 0.0 to 15.0%) in Tris–HCl (pH 8.0) buffer on the purification apparatus (AKTA system, GE-Healthcare-Japan, Tokyo). Subsequently, the eluent was recovered and submitted to dialysis against 20 mM Tris–HCl/150 mM NaCl buffer (pH 8.5) for approximately 17 h, followed by concentrating the solution using Amicon ultra10000 (Millipore, USA). To bring the solution to 50 mM, EDTA was added to it and allowed to react for 1 h at 4 °C, followed by dialysis twice against 2 L of phosphate-buffered saline (PBS). After confirming complete Cu(II) removal via UV/VIS spectroscopy, it was filtered using a 0.2 µm membrane filter (Merck-Millipore) and stored at 4 °C or frozen. Protein concentrations were determined via the Bradford method using the Lowry method with a DC Protein Assay Kit (Bio-Rad).

#### Peptidase activity tests

Most glassware, plasticware, and buffer solutions used were sterilized by heating (180 °C; 2 h), autoclaving (121 °C; 20 min), or filtration through a 0.20-μm sterilized filter, as much as possible to avoid contamination in cleavage assays. Most experiments were performed in a biological safety cabinet to avoid airborne contamination.

FRET-HA peptide (25 µM) was incubated with the antibody samples (5 µM) in 50 mM/tris-100 mM/glycine-Tween-20 (TGT) buffer containing 0.02% NaN_3_ at 37 °C. Fluorescence was measured periodically on the Fluoroskan Ascent (λ_ex_ = 320 nm and λ_em_ = 405 nm; Thermo Scientific Oy, Vantaa, Finland). All measurements were performed in duplicates.

#### Kinetic study

The concentrations of InfA-3L/P95(−), InfA-6L/P95(−), and InfA-9L/P95(−) were fixed at 5 μM and that of the FRET-HA peptide varied from 5 to 60 μM at 37 °C in the TGT buffer (pH 7.7). The initial rate of the reaction included the concentration change of the FRET-HA peptide within 10% conversion after mixing the mutated light chain and the substrate.

#### ELISA

Fifty microliters of an antigen dissolved in PBS solution (5 μg/ml) was fixed on a 96-well plate (Thermo Scientific, Denmark) at 4 °C overnight. Blocking was performed using 100 μL of 2% gelatin and 0.01% thimerosal in PBS for 30 min at room temperature. After washing the plate with phosphate-buffered saline with Tween 20, the antibody sample was immunoreacted, followed by a reaction with POD-conjugated goat affinity-purified antibody to mouse IgG(Fab) (Sigma, A9917-1ML, Lot.0000128663, US). After the substrate reaction was performed with 0.01% H_2_O_2_, which included *o*-phenylenediaminphosphate:2HCl (Wako) dissolved in a 0.1 M citric acid/0.2 M disodium hydrogenphosphate buffer (pH 5.0), the reaction was stopped using 2N H_2_SO_4_. The absorption bands at 490 nm (with 620 nm as the reference) were subsequently measured using a 96-well plate reader (Thermo Scientific, Multiskan, FC).

#### Molecular modeling

Molecular modeling is not appropriate for obtaining a highly accurate understanding. However, it is a useful tool for interpreting the present results without X-ray diffraction analysis.

The deduced antibody light-chain amino acid sequences were used for computational analysis of the antibody structures using Discovery Studio (Accelrys, Inc., San Diego, CA, USA). For homology modeling, template structures were created using a BLAST search, following minimization of the total energy of the molecule using the CHARMM algorithm. The resulting Protein Data Bank data were used to modify the complementarity-determining region (CDR) structures defined using the Kabat numbering system.

#### In vitro assay

The influenza virus used was the A/Hiroshima/37/2001 (H1N1). The virus was grown in an MDCK2 cell culturing medium and harvested and stored as infectious culture fluid at − 152 °C.

MDCK cells were grown in Dulbecco’s modified Eagle’s medium (DMEM) supplemented with 10% fetal bovine serum.

Neutralization tests were performed as follows^40^: The samples diluted with PBS were mixed with an equal volume (150 μL) of influenza virus diluted with DMEM (including 1% BSA) adjusted to give a final control count of about 2000 pfu/ml. After incubation for 48 h at 25 °C, an infectious virus titer of the mixture was calculated using a plaque assay. Each mixture was diluted to 1/2 using DMEM containing Acetyl trypsin (1.5 µg/mL). Then, 0.1 ml of the mixture was inoculated into the MDCK cell monolayer, which was seeded on a 6-well tissue culture tray (Falcon 3046; BD Biosciences). After adsorption for 60 min at 34 °C, the inoculum in each well was removed. The MDCK cells were covered with the first overlay DMEM containing 1.0% agarose ME (Iwai Chemical Industries, Tokyo, Japan) and 1.5 μg/ml acetyl trypsin (Sigma), and the trays were incubated for 2 d in a humidified 5% CO_2_ incubator at 34 °C. After incubation, the cells were covered with a second overlay of DMEM (50 μg/mL neutral red in the first overlay medium). Plaques were counted on the following day.

### Statistical analysis

Statistical analyses were performed using Microsoft Excel for Mac version 16.66.1 for correlation and standard deviation analyses.

### Reporting summary

Further information on the research design is available in the Nature Portfolio Reporting Summary linked to this article.

### Supplementary Information

Supplementary Figures.

## Data Availability

All data required to evaluate the conclusions of this study are presented in the paper and/or Supplementary Materials. Additional data related to this study can be obtained from the corresponding author upon reasonable request. DNA data sets of InfA series antibodies and the mutants generated and/or analyzed during the current study are available in the [DDBJ/GenBank/EMBL] repository (http://getentry.ddbj.nig.ac.jp/). The [accession numbers] of InfA-3/wt, InfA-3/P95(−), InfA-6/wt, InfA-6/P95(−), InfA-9/wt, and InfA-9/P95(−) are [LC767451], [LC767452], [LC767453], [LC767454], [LC767455], and [LC767456], respectively.

## Abbreviations

aa: Amino acid
AMC: 7-Amino-4-methylcoumarin
au: Arbitary unit
CBB: Coomassie brilliant blue
CDR: Complementarity-determining region
DNP: 2,4-Dinitrophenyl
eq: Equivalent
FRET: Förster resonance energy transfer
Fu: Fluorescence unit
HA: Hemagglutinin molecule
IPTG: Isopropyl-β-d-thiogalactopyranosid
mAb: Monoclonal antibody
7-MCA: (7-Methoxycoumarin-4-yl)acetyl
MS: Mass spectroscopy
PBS: Phosphate-buffered saline
SDS–PAGE: Sodium dodecyl sulfate–polyacrylamide gel electrophoresis
TGT: 50 MM/tris-100 mM/glycine-Tween-20 buffer
TFA: Trifluoroacetic acid
VL: Variable region of light chain
