# Supplementary material for: Enzymatization of mouse monoclonal antibodies to the corresponding catalytic antibodies
Source: Sci Rep. 2024 May 28;14:12184. doi: 10.1038/s41598-024-63116-6 (PMC11133420; doi:10.1038/s41598-024-63116-6)
Supplement: Supplementary file 1 — Supplementary Figures. [file 41598_2024_63116_MOESM1_ESM.pdf]

# Supplemental Figures

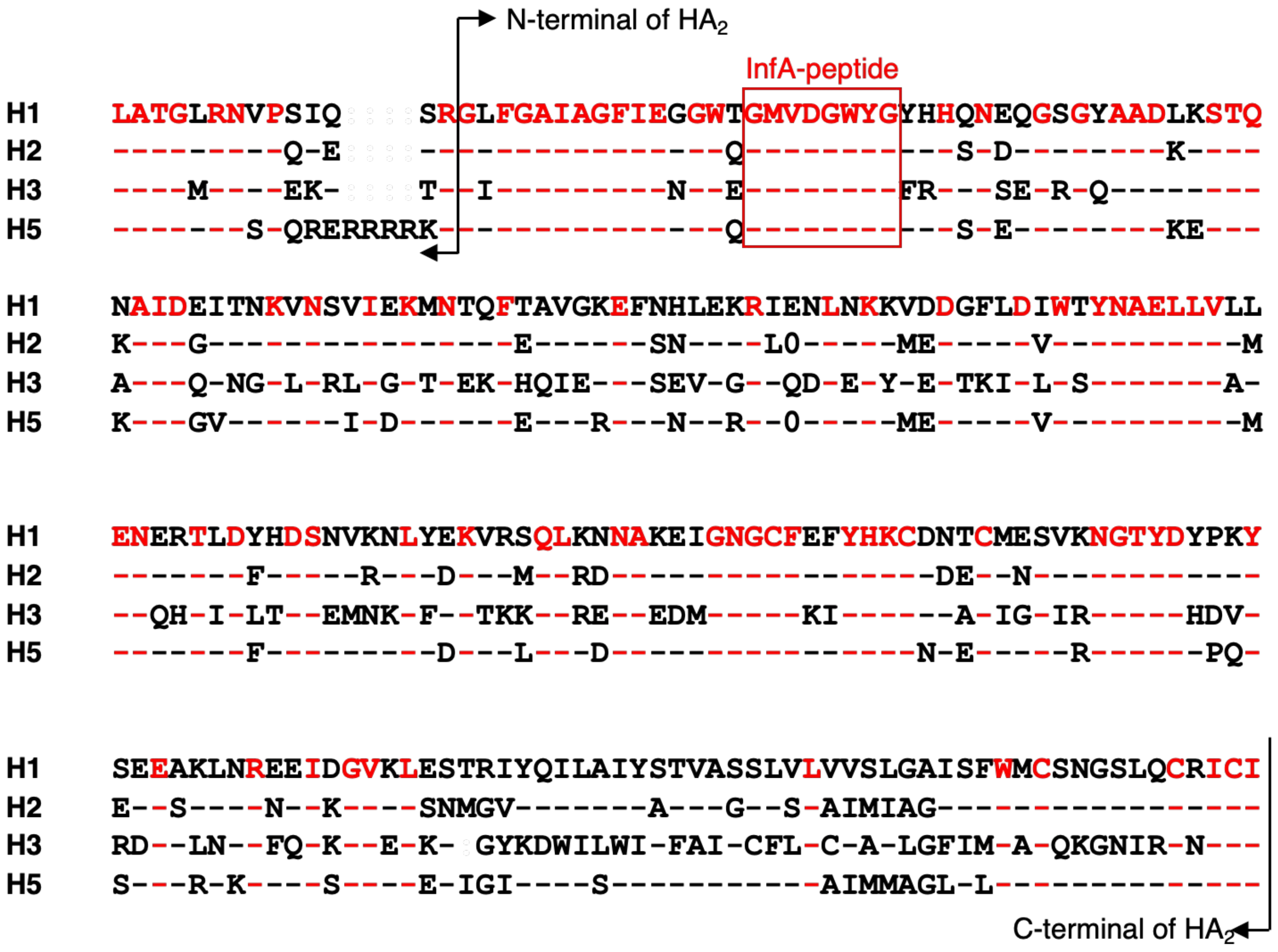


**Supplementary Fig. S1: The aa sequence alignment for the $HA_2$ subunit of the hemagglutinin molecule of the representative four influenza A virus subtypes.** Several conservative peptidyl sequences exist. Herein, the red rectangle region was designated. The peptide (GMVDGWYG) was synthesized and conjugated with bovine serum albumin, which was immunized into Balb/c mice to produce the InfA-series (3-, -6, and -9) monoclonal antibodies[29].

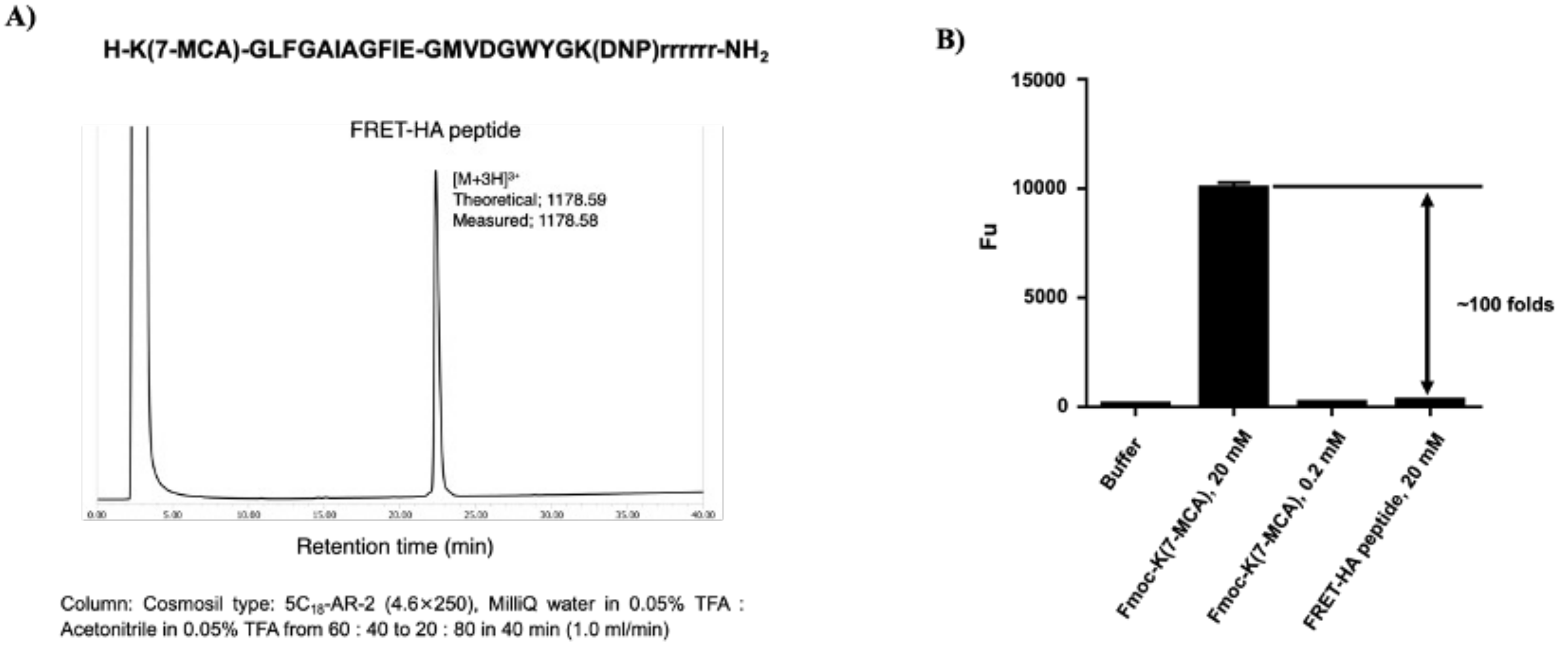


**Supplementary Fig. S2A and S2B:**

**A) Chromatogram of the synthesized FRET-HA peptide**

Column: Cosmosil type: $5C_{18}$-AR-2 (4.6 × 250), MilliQ water in 0.05% TFA: Acetonitrile in 0.05% TFA from 60:40 to 20:80 in 40 min (1.0 ml/min). A peak was detected at 23.5 min, which was $[M+3H]^{3+}$ via HPLC and MS analysis. Its mass was 1178.58, and its theoretical mass was 1178.59.

**B) Quenching performance of the FRET-HA peptide**

The quenching rate (Fmoc-K(7-MCA)/20 mM vs. FRET-HA peptide/20 mM) reached approximately 100 folds. This is sufficient for use in the experiments.

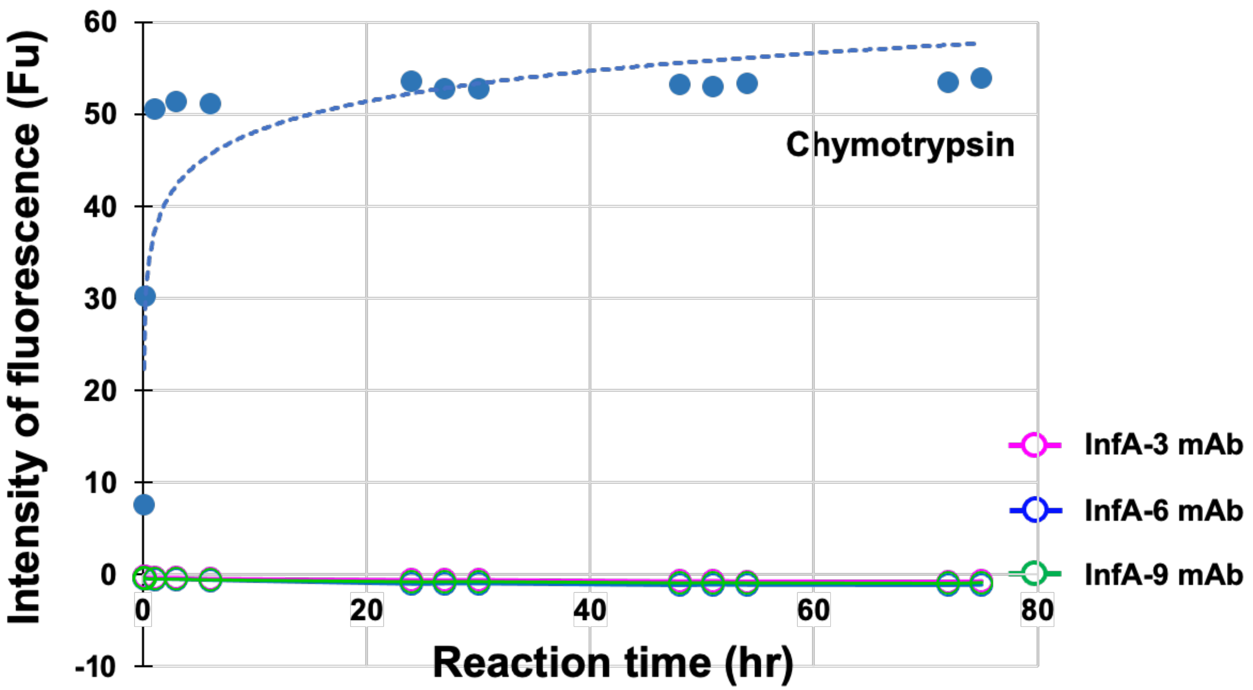


**Supplementary Fig. S3:**
**Time course of the cleavage reaction of FRET-HA peptide by monoclonal antibodies, InfA-3, -6 and -9.**

FRET-HA peptide; 25 µM, mAb; 2.5 µM

The cleavage assay was correctly performed. Any mAbs did not exhibit the catalytic activity.

## Derivation of K value from ELISA

In a heterogeneous reaction using an immunoplate, the immunoreaction of the antibody to the fixed antigen is considered to follow the isothermal adsorption equation proposed by Langmuir-Hinshelwood, which is:

$\theta = KC(1 + KC)$

Here, θis the rate of the fixed antigen that immunoreacted with the antibody, K is the equilibrium constant, and C is the concentration of the antibody. If the rate of the fixed antigen that reacted with the antibody reaches 1/2 (one half of the fixed antigen on the surface of the immunoplate, which reacted with the antibody), the equation becomes:

$1/2 = KC(1 + KC)$

therefore, $K = 1/C$.

When we employ the half-maximal binding value from ELISA (1/2 OD), the reverse value of the concentration of the antibody represents the equilibrium constant (i.e., apparent affinity constant).

Example;
Measurement of K value of InfA-6 mAb (Fig. 4B in main text)

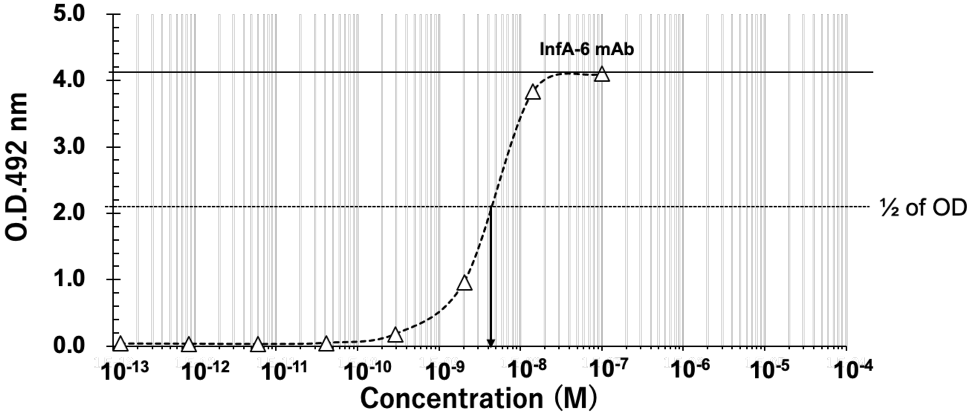


C= 4.1 x $10^{-9}$ M

K=1/C= 1/(4.1 x $10^{-9}$ M)=2.4 x $10^{-8}$/M

**Supplementary Fig. S4:**
Derivation method of affinity constant (K) from ELISA

InfA-18L/wt

DIVMTQSHKFMSTSVGDRVSITCKASQAVGTAV
AWYQQKPGQSPKLLIYWASTRHTGVPDRFTGSG
SGTDFTLTISNVQSEDLADYFCQQYSSYPLTFG
SGTKLEIKRADAAPTVSIFPPSSEQLTSGGASV
VCFLNNFYPKDINVKWKIDGSERQNGVLNSWTD
QDSKDSTYSMSSTLTLTKDEYERHNSYTCEATH
KTSTSPIVKSFNRNEC

InfA-18L/P95(-)

DIVMTQSHKFMSTSVGDRVSITCKASQAVGTAV
AWYQQKPGQSPKLLIYWASTRHTGVPDRFTGSG
SGTDFTLTISNVQSEDLADYFCQQYSSYLTFGS
GTKLEIKRADAAPTVSIFPPSSEQLTSGGASVV
CFLNNFYPKDINVKWKIDGSERQNGVLNSWTDQ
DSKDSTYSMSSTLTLTKDEYERHNSYTCEATHK
TSTSPIVKSFNRNEC

Red: CDR-1 Blue: CDR-2 Green:CDR-3

**Supplementary Fig. S5:**
**Amino acid sequence of InfA-18L/wt and InfA-18L/P95(-)**
InfA-18L belongs to category IGKV6-23*01 of the germline gene, which does not have an appropriate catalytic triad composed of Asp, Ser, and His residues. Therefore, InfA-18L/wt did not exhibit catalytic activity to cleave the HA peptide and a neutralization effect on the viral infection (n = 4). The Pro95-deleted mutant, InfA-18L/P95(-), exhibited the same results as those obtained for InfA-18L/wt because the catalytic site was absent.
